# SIRT3 Regulates HMGCS2 Deacetylation and Influences Cholangiocarcinoma Progression via the Metabolism of Ketone Bodies

**DOI:** 10.1155/humu/9005232

**Published:** 2026-04-07

**Authors:** Sihua Liu, Xiao You, Dongdong Wang, Xin Wang, Yuhang Yang, Fangfang Chen, Juan Zheng, Feiyu Qi, Wanliang Sun, Wei Peng, Jin Xi, Zheng Lu, Dengyong Zhang

**Affiliations:** ^1^ Department of General Surgery, The First Affiliated Hospital of Bengbu Medical University, Bengbu, Anhui, China, bbmc.edu.cn; ^2^ Anhui Key Laboratory of Tissue Transplantation, Bengbu Medical University, Bengbu, Anhui, China, bbmc.edu.cn; ^3^ Department of General Surgery, The Hospital of Fengyang County, Chuzhou, China

**Keywords:** acetylation, cholangiocarcinoma, HMGCS2, ketone bodies, mutation, SIRT3

## Abstract

Cholangiocarcinoma (CCA) is a highly aggressive malignancy. 3‐Hydroxy‐3‐methylglutaryl‐CoA synthase 2 (HMGCS2), a mitochondrial enzyme involved in ketogenesis, has been linked to tumor progression, but its role in CCA remains unclear. HMGCS2 expression in CCA tissues was analyzed using TCGA data and immunoblotting (IB). Functional assays were performed in CCA cell lines (HuCCT‐1 and RBE) and an in vivo xenograft model. Metabolomics explored HMGCS2‐mediated metabolic changes. SIRT3–HMGCS2 interactions were examined via molecular docking, IF, CO‐IP, GST pull‐down, and CHX assays, with mutational analysis identifying interaction sites. IHC assessed clinical samples. HMGCS2 was downregulated in CCA. Overexpression inhibited proliferation and invasion, while knockdown promoted these effects, consistent in vitro and in vivo. Metabolomics showed HMGCS2 enhanced ketone body synthesis, and exogenous ketone bodies mimicked its antitumor effects. SIRT3 deacetylated HMGCS2 at K310 (with plasmid mutation assay), and low HMGCS2/SIRT3 expression correlated with poor patient survival. SIRT3‐mediated deacetylation of HMGCS2 promotes ketone body synthesis, suppressing CCA progression. HMGCS2 is a potential therapeutic target for CCA.

## 1. Introduction

Cholangiocarcinoma (CCA), a rare and highly heterogeneous malignant tumor originating from the bile duct epithelium, not only presents with limited obvious symptoms in the early stages but also lacks an effective diagnostic marker. Owing to its highly invasive nature, CCA has been associated with late patient diagnoses, lack of effective treatments, and a poor prognosis despite radiotherapy (RT) and chemotherapy, with a 5‐year survival rate (SR) of only 7%–20% [1–3]. Energy metabolism variations between normal and cancer cells have recently provided novel therapeutic insights [4, 5]. Tumor cells undergo metabolic reprogramming to meet the energy demands for rapid proliferation, as evidenced by increased glycolysis and fatty acid metabolism. In this regard, it is noteworthy that ketone bodies are fatty acid metabolites and that their relationship with tumor progression is well documented [6]. For instance, ketone bodies induced metabolic changes in pancreatic cancer cells, inhibiting tumor growth and inducing apoptosis [7]. This phenomenon highlights the significance of a deeper understanding of the targets and roles of metabolic reprogramming in tumor progression. In other words, it could provide novel therapeutic insights into CCA management.

The 3‐hydroxy‐3‐methylglutaryl‐CoA synthase 2 (HMGCS2) enzyme, a member of the HMG‐CoA synthase family, functions as a rate‐limiting enzyme for ketogenic reactions and participates in mitochondrion maturation and metabolic reprogramming [8]. According to research, HMGCS2 binds to acetyl‐CoA to form acetoacetyl‐CoA, which is converted to HMG‐CoA by HMG‐CoA reductase (HMGCR) and eventually metabolized to acetoacetate [9]. Various cancer tissues have shown HMGCS2 downregulation, highlighting its potential anticancer effects. For instance, HMGCS2 downregulation was reported in renal clear cell carcinoma cases, with HMGCS2 overexpression potentially inhibiting cancer cell proliferation and glycolysis [10]. Additionally, HMGCS2 has been linked closely to the tumor microenvironment (TME), as well as tumor stemness and immune cell infiltration [11–13]. Furthermore, HMGCS2 has been associated with the clinical parameters of hepatocellular carcinoma (HCC), with its low expression reported as a marker of poor prognosis in HCC [14, 15]. However, the specific role of HMGCS2 in CCA remains unclear.

Protein posttranslational modifications (PTMs) are essential for cellular homeostasis [16]. The PTM process encompasses acetylation, ubiquitination, and phosphorylation. Sirtuin 3 (SIRT3), a nicotinamide adenine dinucleotide (NAD)–dependent protein deacetylase, regulates multiple metabolic pathways in the mitochondrion, including glycolysis, respiration, fatty acid oxidation, and reactive oxidative species (ROS) synthesis [17]. Notably, SIRT3 has been associated with various cancers, and its deletion could lead to metabolic reprogramming and promote tumorigenesis. Furthermore, in CCA cases, SIRT3 could inhibit the Warburg effect via hypoxia‐inducible factor‐1*α* (HIF‐1‐*α*) downregulation, thus preventing tumor progression.

We hypothesized that in CCA, SIRT3‐mediated deacetylation of HMGCS2 at a specific site enhances its enzymatic activity, increases ketone body production, and thereby suppresses tumor progression. Then, we take some assays to verify the function of the SIRT3–HMGCS2‐ketone body axis in CCA.

## 2. Results

### 2.1. CCA Cells Exhibited Low HMGCS2 Expression

Firstly, in order to find out the differential protein expression in CCA, the protein mass spectrometry analysis was performed on CCA samples. Compared to adjacent nontumor tissues, CCA tissues showed significantly lower HMGCS2 protein expression levels (Figure 1a). Furthermore, CCA tissues showed significantly reduced HMGCS2 mRNA levels, according to the results of the analysis of CCA‐related transcriptomics data from the TCGA database (Figure 1b). Additionally, immunoblot (IB) analyses of the collected clinical samples revealed that CCA tissues exhibited significantly lower HMGCS2 levels than adjacent nontumor tissues (Figure 1c).

Figure 1The expression profile of HMGCS2 in CCA. (a) Protein mass spectrometry heatmap analysis of HMGCS2 expression in CCA tissue (CCA [*n* = 8] vs. paracancerous [*n* = 8]). (b) Analysis of low expression of HMGCS2 in CCA in the TCGA database (tumor vs. normal). (c) Measurement of HMGCS2 expression in CCA tissue and paracancerous tissues by immunoblot. P, paracancerous; A, CCA.  ^∗^ *p* < 0.05.

**Figure:**
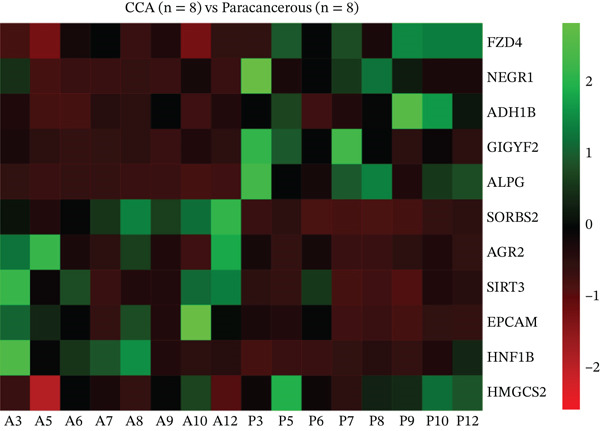
(a)

**Figure:**
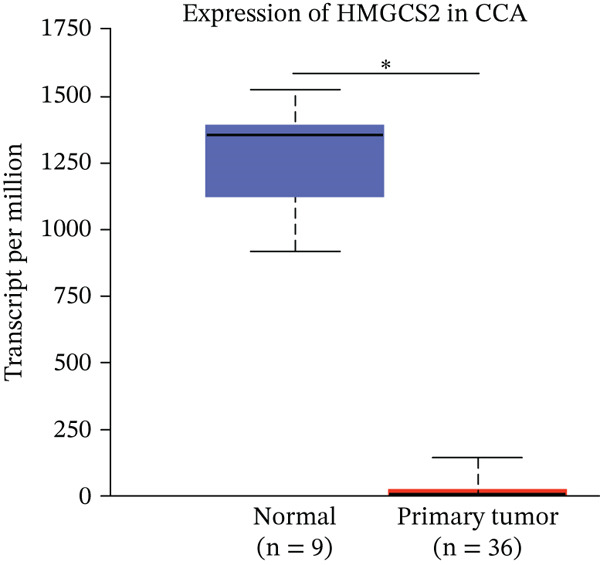
(b)

**Figure:**
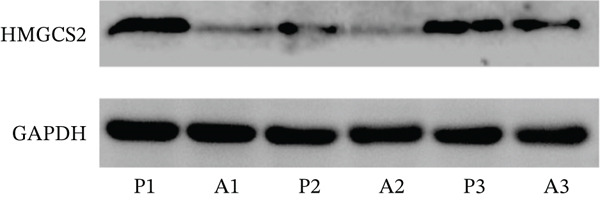
(c)

### 2.2. HMGCS2 Overexpression Inhibited CCA Cell Proliferation and Invasion

Then, we want to explore the biological function of HMGCS2 in CCA cells. We find that HMGCS2 expression was highest in RBE and lowest in the HuCCT‐1 cell line, respectively (Figure 2a). Subsequently, stable CCA cell lines with HMGCS2 knockdown and overexpression were constructed, and the successful construction of these cell models was verified through IB and real‐time fluorescence quantitative PCR (qRT‐PCR) (Figure 2b,c). According to the CCK8 test results, HMGCS2 overexpression inhibited cell proliferation, whereas HMGCS2 knockdown significantly promoted cell proliferation (Figure 2d). Furthermore, according to the colony formation assay results, HMGCS2 overexpression inhibited cell colony formation, whereas HMGCS2 knockdown significantly promoted colony formation (Figure 2e). Finally, according to the Transwell test results, HMGCS2 overexpression inhibited cell invasion, whereas HMGCS2 knockdown significantly facilitated cell invasion (Figure 2f). Also, HMGCS2 overexpression inhibited the tumorigenic ability of animals (Figure 2g).

Figure 2The expression profile of HMGCS2 in CCA cells influencing cell proliferation and invasion. (a) Measurement of HMGCA2 expression in different CCA cell lines by immunoblot. (b, c) Confirmation of the expression levels of HMGCS2 in HMGCS2‐sh and HMGCS2‐OE cells after lentiviral infection by immunoblot and qRT‐PCR. (d) Assessment of the cell proliferation rate by CCK8 assay in HMGCS2‐OE and HMGCS2‐sh group. (e) The effect of HMGCS2 on cell proliferation was determined by the colony formation assay in the HMGCS2‐OE and HMGCS2‐sh groups. (f) The cell migration and invasion were assessed by Transwell assay in the HMGCS2‐OE and HMGCS2‐sh groups (*n* = 3). (g) Animal experiments have studied the tumor proliferation ability in the HMGCS2‐OE group (*n* = 5). Data are presented as the mean ± SD for three independent experiments.  ^∗^ *p* < 0.05,  ^∗∗^ *p* < 0.01, and  ^∗∗∗^ *p* < 0.001.

**Figure:**
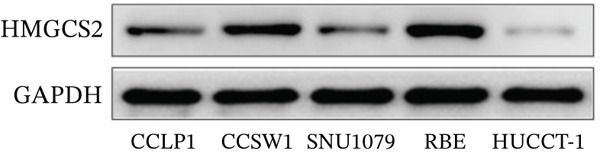
(a)

**Figure:**
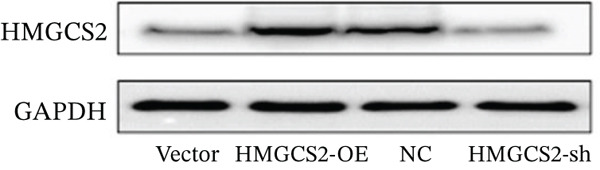
(b)

**Figure:**
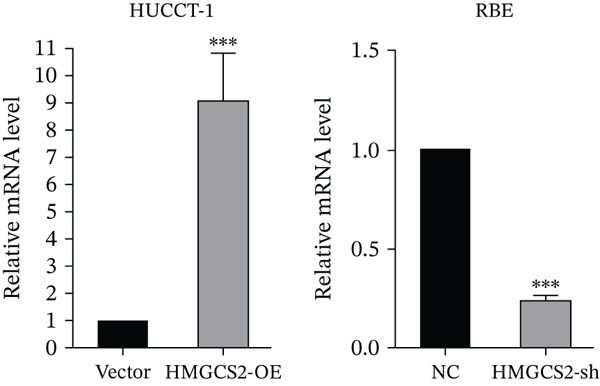
(c)

**Figure:**
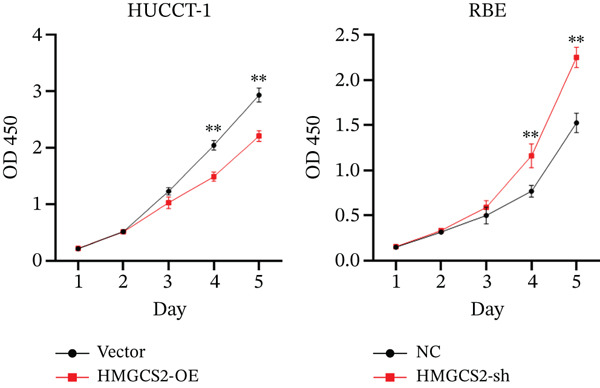
(d)

**Figure:**
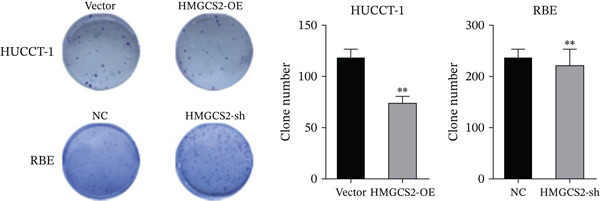
(e)

**Figure:**
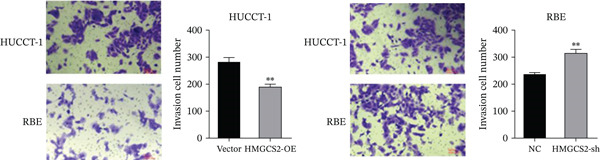
(f)

**Figure:**
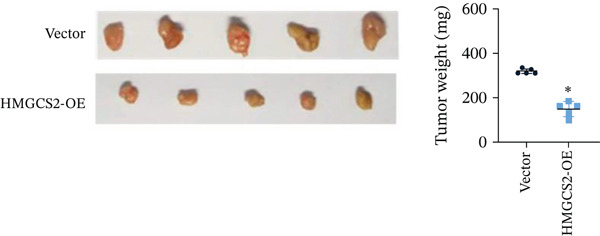
(g)

To verify the effect of HMGCS2 on cell proliferation in vivo, a nude mouse xenograft model was constructed. Specifically, stable HMGCS2 overexpression cells and their control groups were implanted subcutaneously into nude mice. According to the results, HMGCS2 overexpression prevented tumor growth in vivo, a phenomenon that was consistent with the in vitro experimental results.

### 2.3. HMGCS2 Inhibited CCA Cell Proliferation and Invasion by Regulating the Synthesis of Ketone Bodies

Generally speaking, the HMGCS2 always works as an enzyme, a rate‐limiting factor that catalyzes the synthesis of ketone bodies [18], and can regulate their production, thus impacting the malignant phenotypes of CCA cells. Herein, metabolomics analysis was performed on cells with HMGCS2 overexpression and a corresponding control group to further explore the regulatory mechanisms of HMGCS2 in CCA progression. According to the results, HMGCS2 overexpression promoted the synthesis of ketone bodies (Figure 3a,b). We also added a ketone body supplement (KET, 100 *μ*M) to the cell culture system to assess its effect on cell proliferation and invasion. Overexpression of HMGCS2 inhibited the proliferation and invasion of CCA cells, and this inhibitory effect was further enhanced by the addition of ketone bodies. In contrast, knockdown of HMGCS2 promoted the proliferation and invasion of CCA cells, and this promoting effect was partially reversed by the addition of ketone bodies (Figures 3c, 3d, and 3e). These findings suggest that HMGCS2 regulates CCA cell proliferation and invasion through a ketone body–dependent mechanism.

Figure 3HMGCS2 regulates CCA progression via targeting the metabolism of ketone bodies. (a) Metabolomics analysis of HuCCT‐1 cells with stable HMGCS2 overexpression (*n* = 3). (b) Changes in ketone body content in metabolomics test results. (c) The cell proliferation rate was calculated using the CCK8 after the addition of extra ketone bodies (100 *μ*M). (d) The effect of ketone bodies on the proliferation of CCA cells was determined through the colony formation assay after the addition of extra ketone bodies (100 *μ*M). (e) After the addition of extra ketone bodies (100 *μ*M), the cell migration and invasion ability of cells were determined using the Transwell test. Data are presented as the mean ± SD for three independent experiments.  ^∗^ *p* < 0.05,  ^∗∗^ *p* < 0.01, and  ^∗∗∗^ *p* < 0.001.

**Figure:**
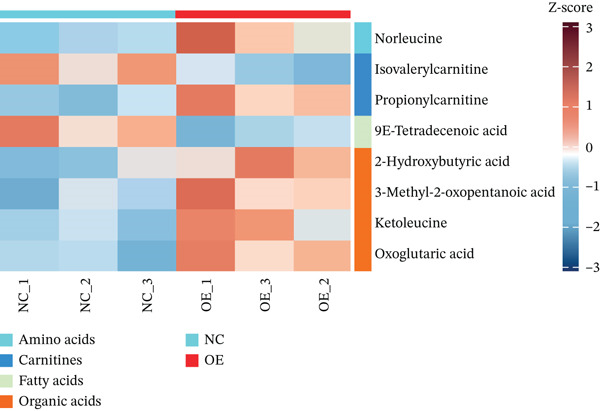
(a)

**Figure:**
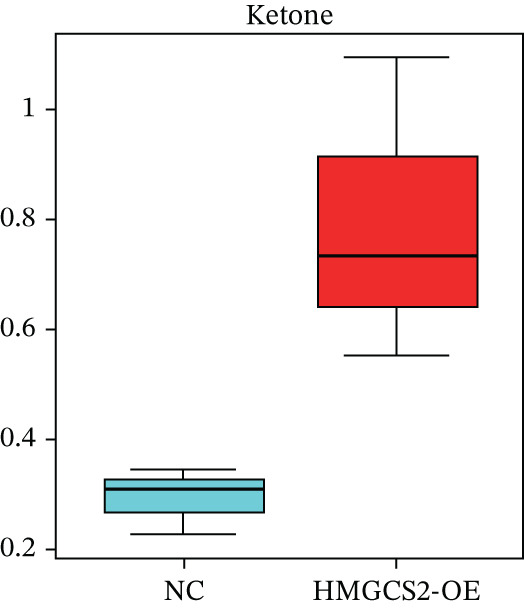
(b)

**Figure:**
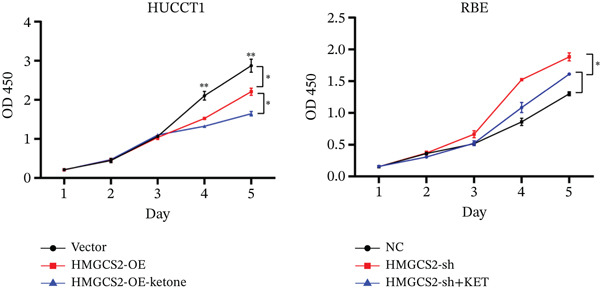
(c)

**Figure:**
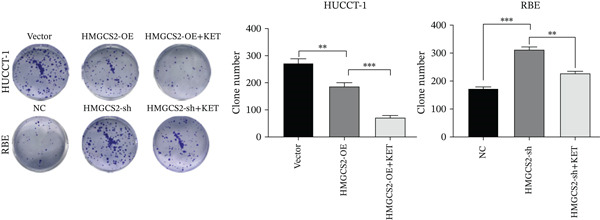
(d)

**Figure:**
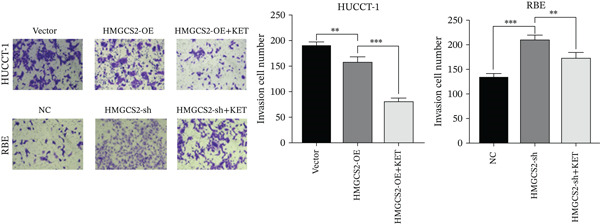
(e)

### 2.4. HMGCS2 Is a Potential Binding Target Molecule for SIRT3

As a protein, it is often regulated by PTMs in cells. The potential of protein PTMs was explored to better understand the regulatory role of HMGCS2. To further establish whether PTMs existed in HMGCS2, PhosphoSitePlus (a proteomics database) was queried. Multiple acetylation sites (Figure 4a) were detected in HMGCS2, including K310, which was highly conserved in different species. According to previous research, SIRT3, an NAD‐dependent deacetylase, is the only member of the sirtuin family primarily localized in the mitochondrion [19, 20]. To assess the HMGCS2‐SIRT3 interaction, the protein structures of the two proteins were first analyzed in the UniProt database, followed by molecular docking analysis using HDOCK. According to the results, the docking scores were high, and hydrogen bonding and hydrophobic interactions were the main interaction forms. These findings strongly suggest that HMGCS2 and SIRT3 may exhibit binding activity (Figure 4b,c).

Figure 4(a) PTM modification site of HMGCS2 in the proteomics database PhosphoSitePlus. (b) Sequence analysis of the conserved K310 region in HMGCS2 orthologs of different species. (c) Using HMGCS2 and SIRT3 as keywords, protein structures were retrieved from UniProt and screened in the database of the human species. The full‐length AlphaFold predicted structures of HMGCSG2_HUMAN (UniProt ID: P54868) and SIR3T_HUMAN (UniProt ID: Q9NTG7) were chosen as the protein structure files. Appropriate docking parameters were set, and protein–protein interaction was performed using HDOCK.

**Figure:**
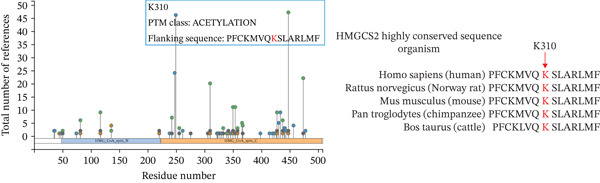
(a)

**Figure:**
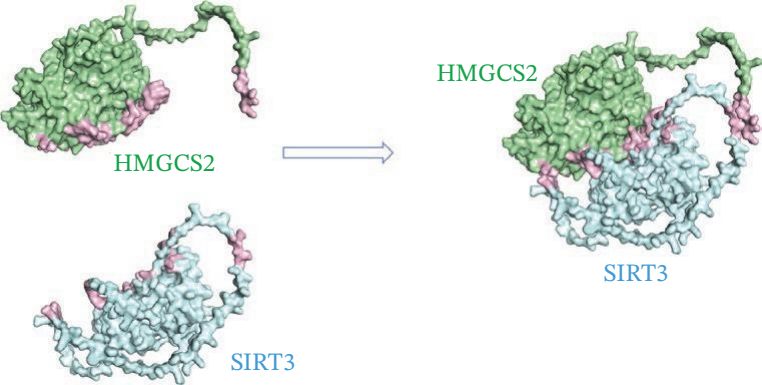
(b)

**Figure:**
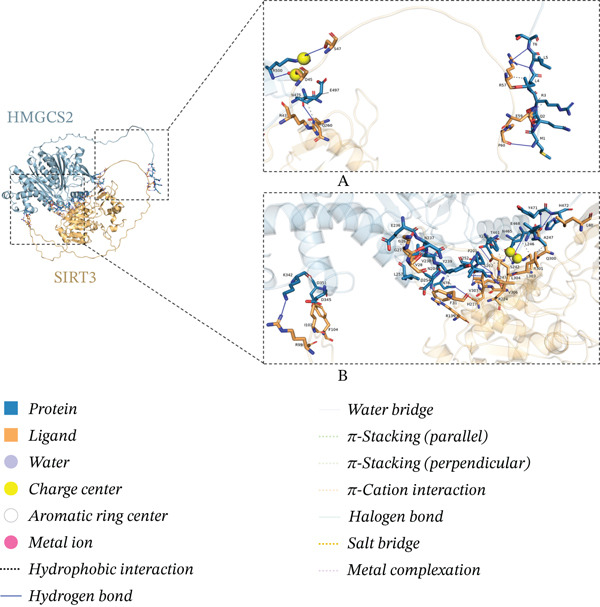
(c)

### 2.5. SIRT3–HMGCS2 Interaction

Herein, coimmunoprecipitation (CO‐IP) experiments were performed to verify the endogenous interaction between HMGCS2 and SIRT3 in cells, revealing that SIRT3 and HMGCS2 could bind to each other (Figure 5a,b). To further establish whether SIRT3 directly interacted with HMGCS2, Myc‐labeled HMGCS2 and HA‐labeled SIRT3 proteins were overexpressed in RBE and HEK‐293T cells. In vivo, interactions between SIRT3 and HMGCS2 were then detected through the CO‐IP assay (Figure 5c,d). The immunofluorescence (IF) staining results showed that the two were primarily localized in the cytoplasm and displayed colocalization (Figure 5e,f). Additionally, the in vitro GST pull‐down assay using recombinant proteins GST‐HMGCS2 and GST‐SIRT3 revealed that GST‐SIRT3 could pull down Myc‐HMGCS2 instead of GST and that HA‐SIRT3 pulled down GST‐HMGCS2 (Figure 5g), further demonstrating the direct HMGCS2‐SIRT3 interaction in vitro.

Figure 5HMGCS2 may be a target molecule of SIRT3. Lysates from (a) RBE and (b) HEK‐293T cells were subjected to the IP analysis using HMGCS2 and SIRT3 antibodies, followed by IB analysis. IgG served as the control isotype. WCL, whole cell lysates. HA‐SIRT3 and Myc‐HMGCS2 were cotransfected into (c) RBE cells and (d) HEK‐293T cells, and lysates were subjected to IP analysis with anti‐HA or anti‐Myc antibodies, followed by IB analysis with anti‐Myc and HA antibodies. (e) Immunofluorescence staining results showing the colocalization of HMGCS2 (green) and SIRT3 (red) in HuCCT‐1 and RBE cells. (f) Pearson′s coefficient of SIRT3 and HMGCS2 in immunofluorescence colocalization. (g) Myc‐HMGCS2 was incubated with purified GST or GST‐SIRT3, and HA‐SIRT3 was incubated with purified GST or GST‐SIRT3. Pulled down with glutathione‐agarose beads. Immunoprecipitation was analyzed by IB.

**Figure:**
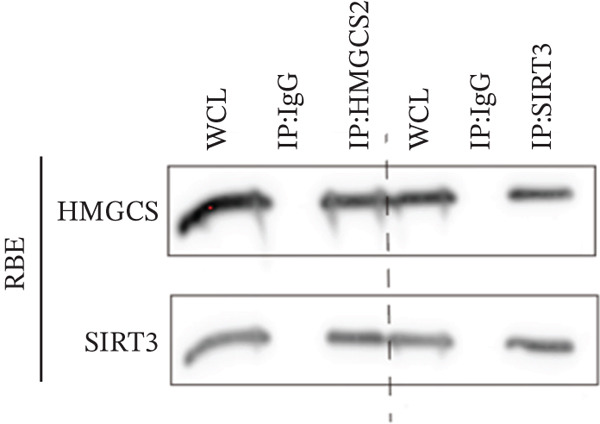
(a)

**Figure:**
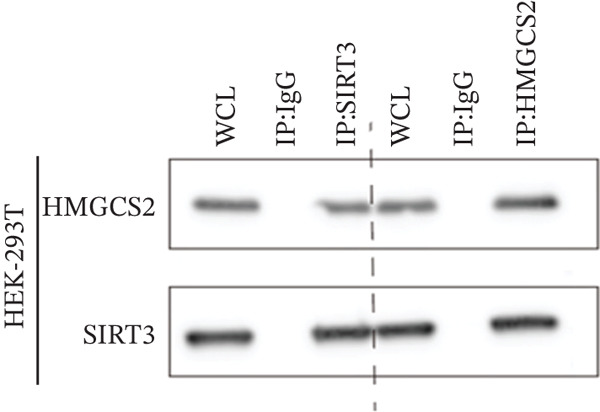
(b)

**Figure:**
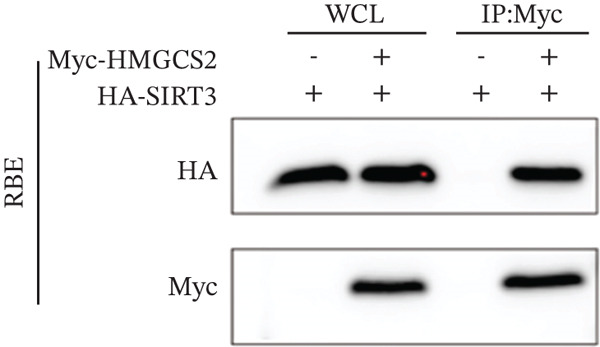
(c)

**Figure:**
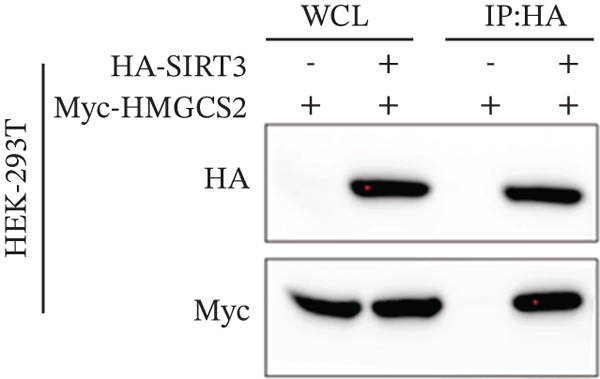
(d)

**Figure:**
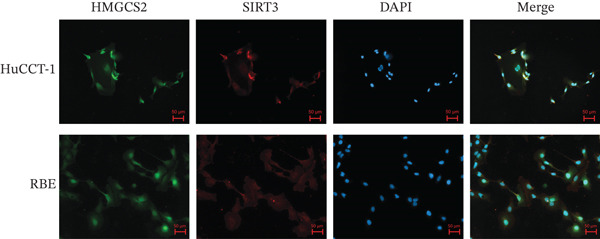
(e)

**Figure:**
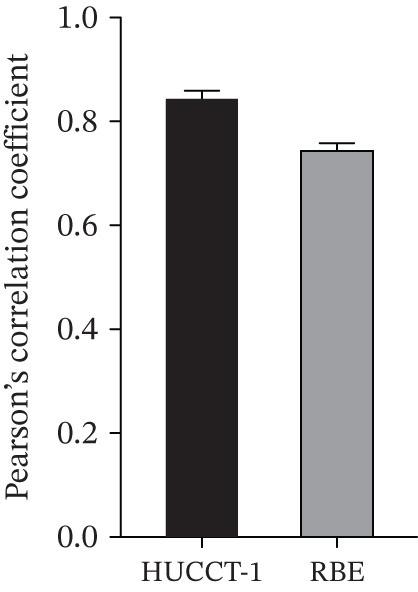
(f)

**Figure:**
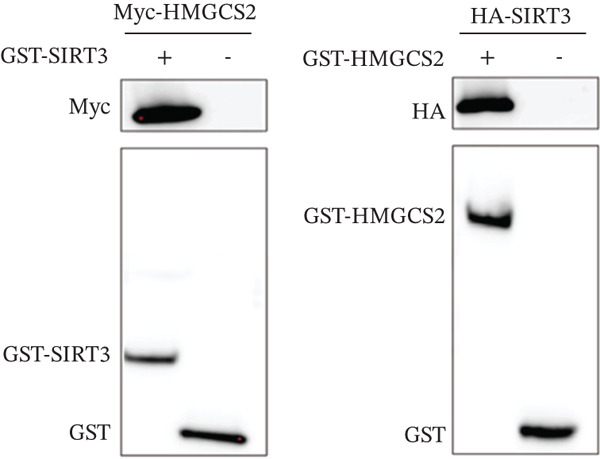
(g)

### 2.6. SIRT3 Regulated HMGCS2 via Deacetylation

To establish whether HMGCS2 was subject to acetylation regulation, Myc‐labeled HMGCS2 proteins were first ectopically expressed in HEK‐293T cells. These cells were then treated with trichostatin A (TSA; an HDAC family deacetylase inhibitor) and nicotinamide (NAM; a SIRT family deacetylase inhibitor). Following that, immunoprecipitation was performed with a pan‐acetylated antibody (Pan‐AC). According to the IB results, HMGCS2 was acetylated, and both TSA and NAM increased its acetylation level, with NAM exerting a significantly greater effect (Figure 6a) dose‐dependently (Figure 6b). This finding suggests that the sirtuin family primarily regulates HMGCS2 acetylation. To further establish whether HMGCS2 deacetylation in the mitochondrion was SIRT3‐induced, Myc‐labeled HMGCS2 and HA‐labeled SIRT3 proteins were ectopically expressed in HEK‐293T cells. According to the IB results, SIRT3 induced HMGCS2 deacetylation (Figure 6c). Subsequently, cycloheximide (CHX) was used to inhibit protein synthesis in stabilized 293T cells with SIRT3 knockdown. According to the results, HMGCS2 expression in the knockdown group increased gradually with the prolongation of CHX action, indicating that SIRT3 knockdown enhanced HMGCS2 protein stability (Figure 6d). Furthermore, upon adding CHX to RBE cells with SIRT3 overexpression, the protein stability of HMGCS2 was significantly degraded (Figure 6e), indicating SIRT3 decreased HMGCS2 protein stability.

Figure 6SIRT3 regulates HMGCS2 protein stability via deacetylation. (a) Myc‐HMGCS2 was transfected into HEK‐293T cells, followed by treatment with TSA (0.5 *μ*M, 16 h) and NAM (5 mM, 4 h). The total cell lysates and anti‐Myc immunoprecipitates were subjected to the IB test. (b) Myc‐HMGCS2 was transfected into RBE cells, followed by incubation with different doses of NAM for 4 h. The total lysates and anti‐Myc immunoprecipitates of the cells were subjected to the IB test. (c) Myc‐HMGCS2 and HA‐SIRT3 were cotransfected into HEK‐293T cells and treated with or without 5 mM NAM for 4 h. The total lysates and anti‐Myc immunoprecipitates of the cells were examined with the IB analysis. (d) HEK‐293T cells stably expressing control shRNA or two independent SIRT3 shRNAs were treated with 50 *μ*g/mL CHX and collected at the indicated times for further IB analysis. (e) RBE cells overexpressing SIRT3 were treated with 50 *μ*g/mL CHX, collected at the indicated times, and subjected to IB analysis using HMGCS2 and SIRT3 antibodies. (f) The total lysates and anti‐Myc immunoprecipitates from HEK‐293T cells transfected with HA‐SIRT3 and Myc‐HMGCS2/K310R were subjected to IB analysis.

**Figure:**
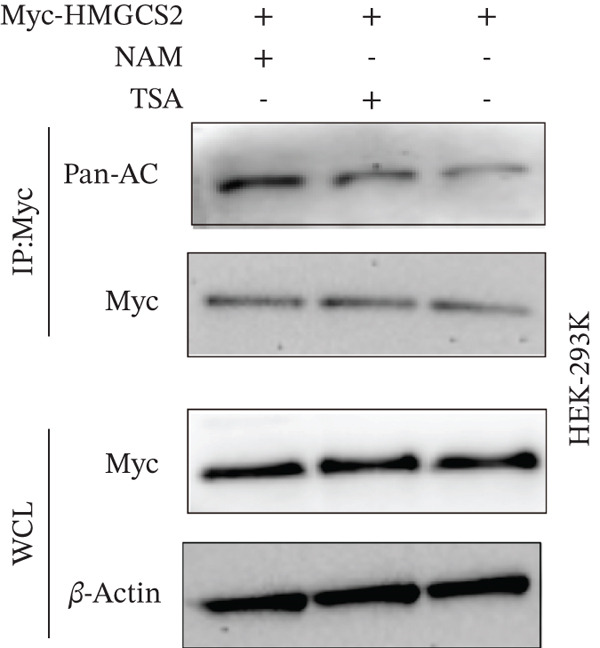
(a)

**Figure:**
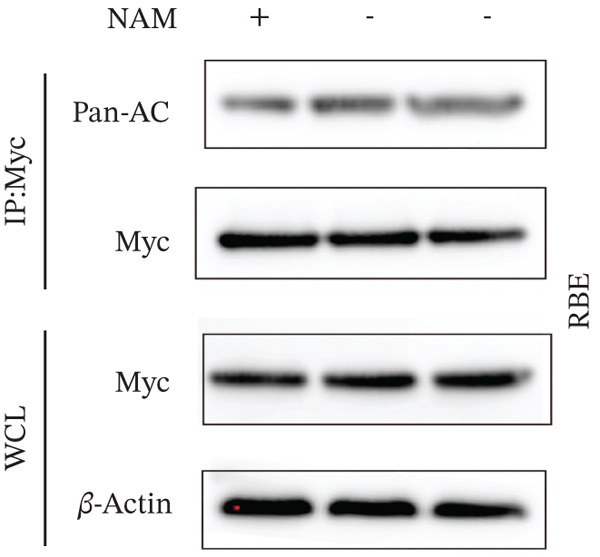
(b)

**Figure:**
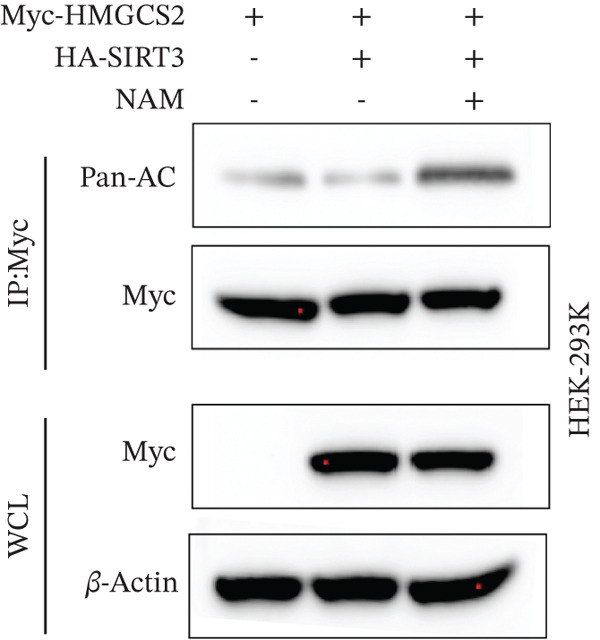
(c)

**Figure:**
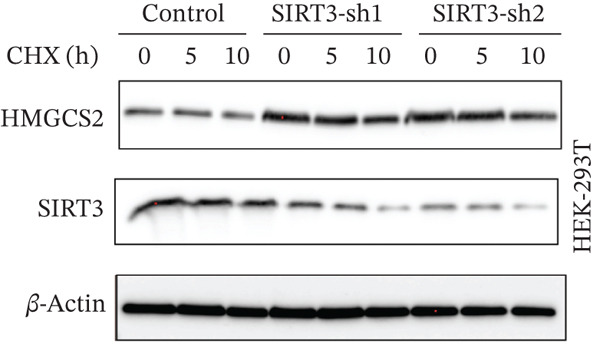
(d)

**Figure:**
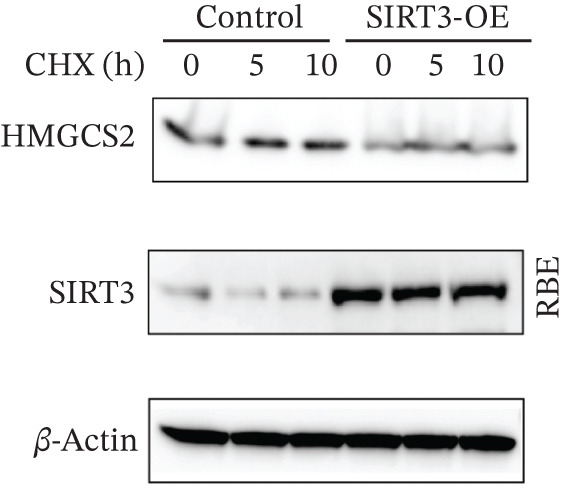
(e)

**Figure:**
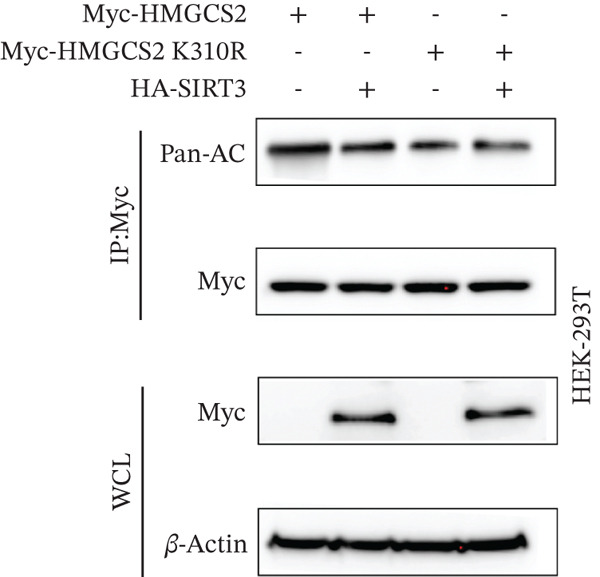
(f)

Subsequently, the binding site between SIRT3 and HMGCS2 was assessed. Based on the analysis of PhosphoSitePlus, we hypothesized that K310 could be the targeting site of SIRT3. To verify this assumption, a mutation of HMGCS2 (K310R) was constructed and coexpressed ectopically with Myc‐HMGCS2 and HA‐SIRT3 proteins in HEK‐293T cells. According to the results, the HMGCS2‐K310R mutation did not lead to an increase in overall acetylation levels even upon SIRT3 overexpression (Figure 6f). This implies that SIRT3 exerted a deacetylation effect by targeting the K310 site on HMGCS2 and then promoted the metabolism of ketone bodies.

### 2.7. SIRT3 and HMGCS2 Correlated With the Clinical Prognosis of CCA

Finally, the relationship between SIRT3 and HMGCS2 protein expression in human CCA samples (*n* = 8) was examined to evaluate the clinical significance of SIRT3 and HMGCS2 in CCA. According to the IB results, SIRT3 and HMGCS2 protein levels were positively correlated in CCA (Figure 7a,b). Furthermore, immunohistochemical staining for HMGCS2 and SIRT3 was carried out on the collected CCA samples (*n* = 65). IHC analysis revealed a significant positive correlation between SIRT3 and HMGCS2 (Figure 7c,d). Additionally, the Kaplan–Meier (KM) survival analysis revealed that the downregulations of HMGCS2 and SIRT3 correlated significantly with decreased overall survival (OS) (Figure 7e,f). Collectively, these results demonstrate that in patients with CCA, SIRT3 modifies HMGCS2 through deacetylation, and both HMGCS2 and SIRT3 are significantly correlated with an unfavorable prognosis.

Figure 7HMGCS2 is lowly expressed in CCA tissues, and its expression is positively correlated with the survival rate of CCA patients. (a) IB analysis of cell lysates from eight CCA tissue samples using HMGCS2 and SIRT3 antibodies. (b) Correlation between HMGCS2 and SIRT3 in CCA tissue samples (*n* = 8). Statistical analysis was conducted using the chi‐squared test. Pearson′s *r* indicates correlation coefficients. (c) Representative images of HMGCS2 and SIRT3 immunohistochemical staining in CCA tissues (*n* = 65). (d) Correlation analysis of IHC scores of HMGCS2 and SIRT3 in CCA tissue samples (*n* = 65). Chi‐squared test was used for statistical analysis. Pearson′s *r* indicates the correlation coefficient. (e) Kaplan–Meier curve showing the overall survival of CCA patients based on HMGCS2 expression in tumors (*p* value was verified by log‐rank test). (f) Kaplan–Meier curve indicating the overall survival of CCA patients based on SIRT3 expression in tumors (*p* value was verified by log‐rank test).

**Figure:**
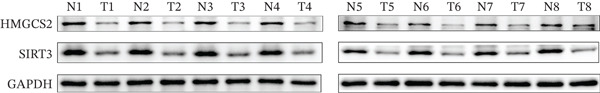
(a)

**Figure:**
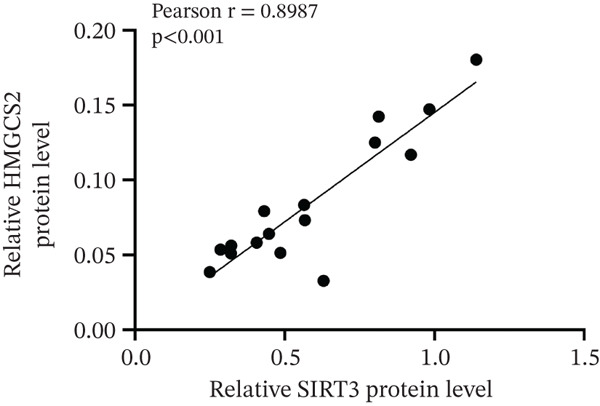
(b)

**Figure:**
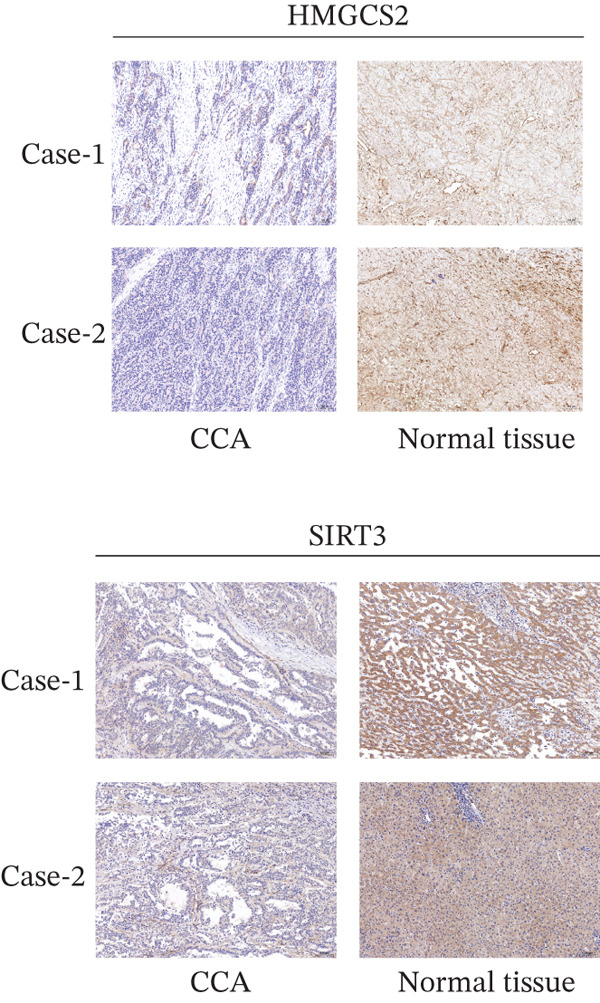
(c)

**Figure:**
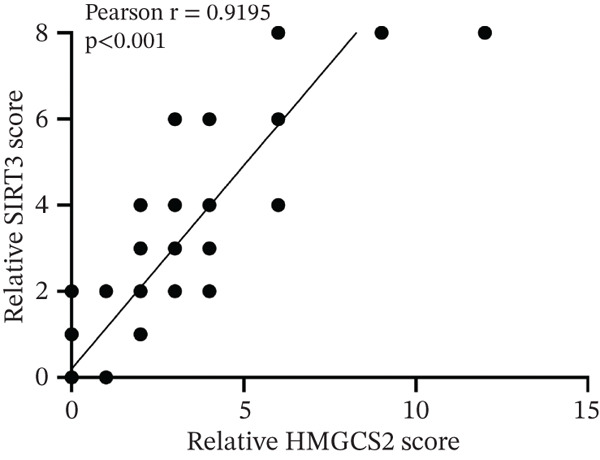
(d)

**Figure:**
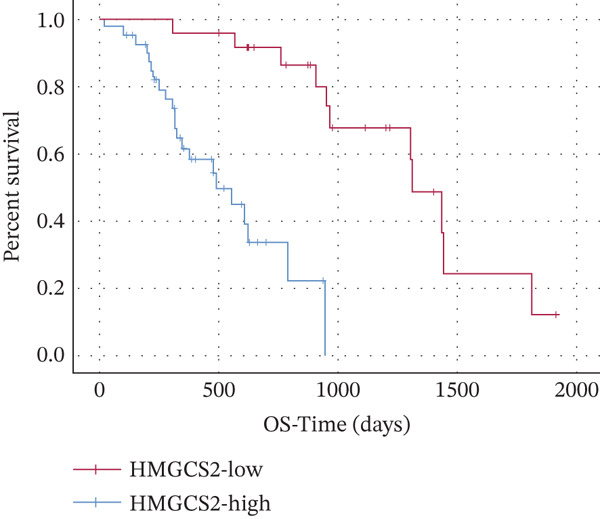
(e)

**Figure:**
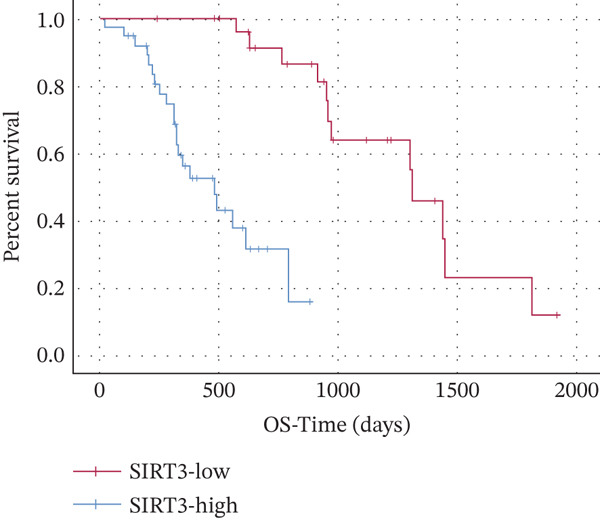
(f)

## 3. Discussion

Our findings in this study supported the notion that changes in HMGCS2 expression in CCA cells could induce a relative increase or decrease in the production of ketone bodies. The ketone bodies increased significantly following HMGCS2 overexpression in CCA cells. Furthermore, the supplementation of ketone bodies partially reversed the HMGCS2 knockdown‐induced enhanced effects of cell proliferation and invasion. Moreover, in an HCC‐related study, ketone body supplementation reduced tumor cell viability and inhibited metastatic cancer cell proliferation and invasion [14]. Therefore, this study provides new evidence suggesting that the metabolism of ketone bodies could inhibit CCA progression via HMGCS2.

Due to its highly invasive characteristics, the majority of CCA patients are asymptomatic in the early stages and lack reliable early diagnostic biomarkers, leading to low long‐term SRs [21]. Metabolic differences between CCA and normal bile duct tissues have recently provided novel insights into targeted therapy for cancer cells [22–24]. Consequently, new biomarkers with higher prognostic or therapeutic values could be identified, bringing more benefits to CCA patients [25, 26]. Energy metabolism is crucially involved in normal cell growth. Tumor cells, due to their rapid proliferation, require energy metabolism reprogramming to adapt to constantly changing energy demands [27–29]. Therefore, energy metabolism reprogramming could be a hallmark feature of tumorigenesis [30]. The HMGCS2 enzyme, a rate‐limiting factor that regulates the production of ketone bodies, is mainly involved in fatty acid catabolism, facilitating energy production in the liver. Whereas HMGCS2 is often silenced in proliferating cells, it has been detected in differentiated cells. For instance, HMGCS2 was previously found to be significantly downregulated in both moderately and poorly differentiated colorectal adenocarcinoma (CRC) cells [12]. Furthermore, our findings revealed that HMGCS2 was lowly expressed in malignant proliferating cells. Additionally, HMGCS2 overexpression significantly inhibits CCA cell proliferation and invasion. Moreover, HMGCS2 upregulation in fenofibrate‐induced melanoma cells was previously found to correlate with proliferation inhibition [31], further confirming the effects of HMGCS2 on cell proliferation.

Ketone bodies, which are metabolites of HMGCS2, have recently gained increasing attention for their crucial roles in metabolic differences between normal and cancer cells [32]. Protein PTMs in metabolism have recently garnered widespread attention for their response to changes in the body′s metabolic state and cellular homeostasis maintenance [33]. Notably, SIRT3, an NAD‐dependent mitochondrial deacetylase, has been established as an essential factor for mitochondrial function optimization. During fasting, SIRT3 may induce HMGCS2 deacetylation, enhancing the ketogenic effect [34]. Considering that the body′s energy metabolism state during tumorigenesis is comparable to that during fasting, it is plausible that SIRT3 may mediate HMGCS2 deacetylation in CCA. This phenomenon is consistent with the results of the present study, which posited that SIRT3 can induce HMGCS2 deacetylation by interacting directly with its K310 site. Furthermore, HMGCS2 deacetylation maintains its high enzymatic activity, further elevating the ketogenic level, thus inhibiting CCA cell proliferation.

Previous research has shown that HMGCS2 expression varies across different types of human cancers and is associated with the prognosis of these malignancies [12, 35, 36]. At the same time, its low expression has been associated with poor prognosis in esophageal squamous cell carcinoma (ESCC) and colon cancer [37]. The same result was found in our study; we revealed that CCA tissues exhibited a significantly lower HMGCS2 and SIRT3 expression than normal bile duct tissues. Additionally, HMGCS2 expression correlated with the survival of CCA patients. These findings collectively suggest that HMGCS2 may exhibit antitumor activity and serve as a potential biomarker in CCA.

In conclusion, our findings revealed the significant potential of SIRT3 and HMGCS2 as novel biomarkers and therapeutic strategies in CCA. Our insights into the SIRT3–HMGCS2‐ketone body axis illuminate the role of deacetylation, energy metabolism, and PTM in CCA. However, the acetylases of HMGCS2 were not identified. In other words, additional research will be required in the future to establish whether other factors (such as ubiquitination modification and succinylation modification) could also regulate HMGCS2.

## 4. Materials and Methods

### 4.1. Cell Culture and Tissue Samples

First, CCA cell lines (RBE, HCCC‐9810, and HuCCT‐1) and human HEK‐293T cells (all from the Cell Bank of the Chinese Academy of Sciences; Shanghai National Identification Cell Culture Bank) were cultured in the Roswell Park Memorial Institute (RPMI 1640) medium (11875093, Gibco) supplemented with 10% fetal bovine serum (FBS, 10099‐141c, Gibco) and a 1% penicillin–streptomycin solution (15070063, Thermo Fisher). The ketone bodies used for cell culture included 78% *β*‐HB, 20% AcAc, and 2% acetone.

Before the experiments, all cell lines were identified through short tandem repeat (STR) profiling and regularly tested for mycoplasma contamination every 3 months using the Mycoplasma Test Kit (J66117, Thermo Fisher). CCA tissue specimens were obtained from the First Affiliated Hospital of Bengbu Medical University under protocols approved by the Institutional Ethics Committee of Bengbu Medical University (Approval No. 20240314).

### 4.2. Plasmid Transfection and Lentivirus Infection

The HA‐SIRT3, Myc‐HMGCS2, and HA‐HMGCS2‐K310R plasmids were constructed using pcDNA3.1 (37680, Addgene), while the GST‐HMGCS2 and GST‐SIRT3 plasmids were constructed using pGEX‐4T‐1 (129567, Addgene). On the other hand, the Myc‐HMGCS2, HA‐SIRT3, and sh‐SIRT3 plasmids were constructed using the Plvx‐Puro vector (Bio‐114923, Biobw). Four plasmid groups (VSVG, pLP1, pLP2, and the target plasmids) were subsequently transfected into HEK‐293T cells using Lipofectamine 3000 to generate the virus. Additionally, CCA or HEK‐293T cell lines were infected with Polybrene (TR‐1003, Sigma‐Aldrich), and stabilized cell lines were identified through puromycin screening.

### 4.3. qRT‐PCR

First, total RNA was extracted from the cells using FreeZol Reagent (R711, Vazyme, China) and then reverse‐transcribed into cDNA using the Reverse Transcription Kit (R323, Vazyme, China). Subsequently, the ChamQ Universal SYBR qPCR Master Mix (Q711, Vazyme, China), cDNA, and primers were mixed proportionally into octuplex tubes for real‐time PCR on QuantStudio3 (Thermo Fisher Science, United States). All operations were performed per the manufacturer′s instructions. See Table 1 for the primer sequences.

**Table 1:** Primer sequences used for fluorescence quantitative real‐time PCR.

Name	Target sequence (5 ^′^–3 ^′^)
HMGCS2	F: 5 ^′^‐AAGTCTCTGGCTCGCCTGATGT‐3 ^′^ R: 5 ^′^‐TCCAGGTCCTTGTTGGTGTAGG‐3 ^′^
SIRT3	F: 5 ^′^‐ACCCAGTGGCATTCCAGAC‐3 ^′^ R: 5 ^′^‐GGCTTGGGGTTGTGAAAGAAG‐3 ^′^
GAPDH	F: 5 ^′^‐TCACCACCATGGAGAAGGC‐3 ^′^ R: 5 ^′^‐GCTAAGCAGTTGGTGGTGCA‐3 ^′^

### 4.4. IB Analysis

First, total cell or tissue proteins were extracted using RIPA lysis buffer (NCM Biotech, China) containing protease and phosphatase inhibitors. Protein concentration was then assessed using the bicinchoninic acid assay (BCA) method (23227, Thermo Fisher, United States). Subsequently, protein samples were separated through sodium‐dodecyl sulfate polyacrylamide gel electrophoresis (SDS‐PAGE) and then transferred onto 0.45 *μ*m polyvinylidene difluoride (PVDF) membranes. The membranes were then blocked with a blocking buffer and incubated with primary antibodies at 4°C overnight. Subsequently, the membranes were incubated with horseradish peroxidase (HRP)–conjugated secondary antibodies for 1 h. Finally, the blot was incubated with the ECL luminescent reagent (WBKLS0500, Immobilon, United States) before capturing images on the Bio‐Rad ChemiDoc system. All tests were independently repeated three times. Table 2 lists the antibodies used for IB analysis.

**Table 2:** The primary and secondary antibodies used in this study are listed.

Antibody	Source	Dilution
HMGCS2	CST	WB:1:1000 and IP:1:50
HMGCS2	Affinity	IF:1:500
SIRT3	CST	WB:1:1000 and IP:1:50
SIRT3	NOVUS	IF:1:500
GAPDH	CST	WB:1:1000
Beta‐actin	CST	WB:1:1000

### 4.5. Colony Formation Experiment

First, the cells were inoculated into six‐well plates at a density of 1000 cells/well. The medium was changed every 3 days. After 2 weeks, the cells were fixed with a 4% paraformaldehyde solution (Biosharp, China) for 15 min, stained with crystal violet (Beyotime, China) for 15 min, and washed with PBS three times before image acquisition.

### 4.6. Transwell Test

First, the matrix gel (356234, BD Biosciences) was diluted with precooled serum‐free medium and then added to the upper chamber of the Transwell, where it was spread evenly on the bottom surface. The Transwell was then incubated for 2 h. Subsequently, 200 *μ*L of serum‐free medium containing 2 × 10^4^ cells was added to the upper chamber, while 600 *μ*L of a medium containing 10% FBS was added to the lower chamber. The Transwell was then incubated for an additional 24 h. Following that, the bottom cells were fixed with a 4% paraformaldehyde solution (Biosharp, China) for 15 min and then stained with crystal violet for 15 min. Finally, the cells were washed three times with PBS before image acquisition.

### 4.7. IF Analysis

First, CCA cells on round coverslips were fixed in a 4% paraformaldehyde solution for 20 min, permeabilized with 0.2% Triton X‐100 (85111, Thermo Fisher) for 5 min, and then blocked with 3% bovine serum albumin (BSA) for 1 h. The cells were then incubated with the indicated primary antibodies at 4°C overnight. Following that, the cells were washed with PBS and then incubated with fluorescent dye‐coupled secondary antibodies for 1 h. Subsequently, the slide was sealed with an antifluorescence burst sealant (containing DAPI) before capturing images using a fluorescence microscope (Axio Observer Z1, ZEISS, Germany).

### 4.8. CO‐IP

First, the cells were lysed with the IP pyrolysis solution. Protein complexes were then captured with anti‐HMGCS2 or anti‐SIRT3 antibodies, as well as immunoglobulin G (IgG), at 4°C overnight. On the following day, the antigen–antibody complexes were captured using protein A + G magnetic beads. After mixing for 2 h at room temperature (RT), the supernatant was discarded to obtain antigen–antibody complexes for IB experiments. The whole‐cell lysate was separated through SDS‐PAGE, and corresponding antibodies were employed for IB detection.

### 4.9. GST Pull‐Down Test

Protein–protein interactions (PPIs) were detected using the GST pull‐down test. First, the Myc‐HMGCS2 and HA‐SIRT3 plasmids were transfected into HEK‐293T cells. Subsequently, the cells were lysed with the GST pyrolysis solution, and proteins were extracted. On the other hand, purified GST‐HMGCS2 or GST‐SIRT3 proteins were extracted from *E. coli* BL21 (DE3, EC0114, Thermo Fisher). Following the manufacturer′s instructions, the proteins were detected and purified using the BeyoGold GST‐tag Resin Purification Kit (P2251, Beyotime). The beads were washed with the GST pull‐down binding buffer, and the purified GST‐SIRT3 was incubated with total protein from Myc‐HMGCS2‐transfected HEK‐293T cells for 4 h at 4°C on a rotary mixer. Notably, GST‐HMGCS2 and HA‐SIRT3 underwent a similar treatment. Finally, the mixture was subjected to IB analysis.

### 4.10. Xenograft Model

Herein, 12 male BALB/c nude mice (age = 6 weeks, weight = 20–25 g) purchased from the Shanghai Laboratory Animal Center were used. All animals were housed in a specific pathogen‐free (SPF) grade animal husbandry facility under a 12/12 h light–dark cycle and controlled temperature conditions, with free access to food and water. The animals were acclimatized for 1 week, after which HuCCT‐1 cells (1.0 × 10^6^) were injected subcutaneously into the left axilla of nude mice. The animals were randomly assigned to two groups: control and HMGCS2‐OE (*N* = 6/group). The tumor volume was measured every 3 days after tumor formation. The tumor volume calculation formula was Volume = (Length × width2)/2. After 4 weeks, the mice were euthanized and tumors were extracted and weighed.

## 5. Statistical Analysis

Cell line experiments were repeated at least three times. Statistical analyses for experimental data were performed using SPSS 25.0 and GraphPad Prism 9.0 software. Results for each group were expressed as mean ± SD (standard deviation). For normally distributed data, means were compared between two groups using the unpaired *t*‐test, while the chi‐square test and one‐way ANOVA were used to compare means between three or more groups. Patients′ SR was assessed using the KM method. The chi‐square test and linear regression analysis were employed to statistically analyze the correlation between HMGCS2 and SIRT3. Results or differences with *p* < 0.05 were considered statistically significant.

## Author Contributions

Conceptualization: Dengyong Zhang and Zheng Lu. Methodology: Dongdong Wang. Software: Wei Peng and Fangfang Chen. Validation: Sihua Liu, Xiao You, and Xin Wang. Formal analysis: Yuhang Yang. Investigation: Feiyu Qi. Resources: Jin Xi. Data curation: Juan Zheng. Writing—original draft preparation: Sihua Liu. Writing—review and editing: Dengyong Zhang. Visualization: Sihua Liu. Supervision: Wei Peng. Project administration: Wanliang Sun and Jin Xi. Funding acquisition: Dongdong Wang, Fangfang Chen, Zheng Lu, and Dengyong Zhang. Sihua Liu, Xiao You, and Dongdong Wang contributed equally to this work.

## Funding

This study was funded by the Natural Science Research Project of Anhui University (2024AH051260, 2023AH052010, 2024AH051282, and 202308085MH271) and the Clinical and Translational Research Project of Anhui Province (02527c10020085).

## Disclosure

All authors have read and agreed to the published version of the manuscript.

## Ethics Statement

This study was approved by the Ethics Committee of Bengbu Medical University (Nos. 2023213 and 2023371).

## Consent

Informed consent was obtained from all subjects involved in the study.

## Conflicts of Interest

The authors declare no conflicts of interest.

## Data Availability

All data relevant to the study are included in this manuscript.

## References

[1] Banales J. M. , Marin J. J. G. , Lamarca A. , Rodrigues P. M. , Khan S. A. , Roberts L. R. , Cardinale V. , Carpino G. , Andersen J. B. , Braconi C. , Calvisi D. F. , Perugorria M. J. , Fabris L. , Boulter L. , Macias R. I. R. , Gaudio E. , Alvaro D. , Gradilone S. A. , Strazzabosco M. , Marzioni M. , and Gores G. J. , Cholangiocarcinoma 2020: The Next Horizon in Mechanisms and Management, Nature Reviews. Gastroenterology ands hepatology. (2020) 17, no. 9, 557–588, 10.1038/s41575-020-0310-z, 32606456. PMC7447603 32606456

[2] Valle J. W. , Kelley R. K. , Nervi B. , Oh D. Y. , and Zhu A. X. , Biliary Tract Cancer, Lancet. (2021) 397, no. 10272, 428–444, 10.1016/S0140-6736(21)00153-7. 33516341

[3] Ilyas S. I. , Khan S. A. , Hallemeier C. L. , Kelley R. K. , and Gores G. J. , Cholangiocarcinoma - Evolving Concepts and Therapeutic Strategies, Nature Reviews. Clinical Oncology. (2018) 15, no. 2, 95–111, 10.1038/nrclinonc.2017.157, 2-s2.0-85040836415, 28994423. PMC5819599 28994423

[4] Kroemer G. and Pouyssegur J. , Tumor Cell Metabolism: Cancer′s Achilles′ Heel, Cancer Cell. (2008) 13, no. 6, 472–482, 10.1016/j.ccr.2008.05.005, 2-s2.0-44449147036. 18538731

[5] Pavlova N. N. and Thompson C. B. , The Emerging Hallmarks of Cancer Metabolism, Cell Metabolism. (2016) 23, no. 1, 27–47, 10.1016/j.cmet.2015.12.006, 2-s2.0-84955326448, 26771115. 26771115 PMC4715268

[6] Qin J. , Huang X. , Gou S. , Zhang S. , Gou Y. , Zhang Q. , Chen H. , Sun L. , Chen M. , Liu D. , Han C. , Tang M. , Feng Z. , Niu S. , Zhao L. , Tu Y. , Liu Z. , Xuan W. , Dai L. , Jia D. , and Xue Y. , Ketogenic Diet Reshapes Cancer Metabolism Through Lysine *β*-hydroxybutyrylation, Metabolism. (2024) 6, no. 8, 1505–1528, 10.1038/s42255-024-01093-w. 39134903

[7] Shukla S. K. , Gebregiworgis T. , Purohit V. , Chaika N. V. , Gunda V. , Radhakrishnan P. , Mehla K. , Pipinos I. I. , Powers R. , Yu F. , and Singh P. K. , Metabolic Reprogramming Induced by Ketone Bodies Diminishes Pancreatic Cancer Cachexia, Cancer and Metabolism. (2014) 2, no. 1, 10.1186/2049-3002-2-18, 25228990. PMC4165433 25228990

[8] Chong D. , Gu Y. , Zhang T. , Xu Y. , Bu D. , Chen Z. , Xu N. , Li L. , Zhu X. , Wang H. , Li Y. , Zheng F. , Wang D. , Li P. , Xu L. , Hu Z. , and Li C. , Neonatal Ketone Body Elevation Regulates Postnatal Heart Development by Promoting Cardiomyocyte Mitochondrial Maturation and Metabolic Reprogramming, Cell Discovery. (2022) 8, no. 1, 10.1038/s41421-022-00447-6, 36220812. PMC9553951 36220812

[9] Puchalska P. and Crawford P. A. , Multi-Dimensional Roles of Ketone Bodies in Fuel Metabolism, Signaling, and Therapeutics, Cell Metabolism. (2017) 25, no. 2, 262–284, 10.1016/j.cmet.2016.12.022, 2-s2.0-85013177882, 28178565. 28178565 PMC5313038

[10] Mao H. , Wang R. , Shao F. , Zhao M. , Tian D. , Xia H. , and Zhao Y. , HMGCS2 Serves as a Potential Biomarker for Inhibition of Renal Clear Cell Carcinoma Growth, Scientific Reports. (2023) 13, no. 1, 14629, 10.1038/s41598-023-41343-7, 37670031. 37670031 PMC10480187

[11] Sun M. , Tan Z. , Lin K. , Li X. , Zhu J. , Zhan L. , and Zheng H. , Advanced Progression for the Heterogeneity and Homeostasis of Intestinal Stem Cells, Stem Cell Reviews and Reports. (2023) 19, no. 7, 2109–2119, 10.1007/s12015-023-10578-2, 37351833. 37351833

[12] Wei R. , Zhou Y. , Li C. , Rychahou P. , Zhang S. , Titlow W. B. , Bauman G. , Wu Y. , Liu J. , Wang C. , Weiss H. L. , Evers B. M. , and Wang Q. , Ketogenesis Attenuates KLF5-Dependent Production of CXCL12 to Overcome the Immunosuppressive Tumor Microenvironment in Colorectal Cancer, Cancer Research. (2022) 82, no. 8, 1575–1588, 10.1158/0008-5472.CAN-21-2778, 35247887. 35247887 PMC9018557

[13] Zhang L. , Shi J. , Du D. , Niu N. , Liu S. , Yang X. , Lu P. , Shen X. , Shi N. , Yao L. , and Zhang R. , Ketogenesis Acts as an Endogenous Protective Programme to Restrain Inflammatory Macrophage Activation During Acute Pancreatitis, EBioMedicine. (2022) 78, 103959, 10.1016/j.ebiom.2022.103959, 35339899. 35339899 PMC8960978

[14] Wang Y. H. , Liu C. L. , Chiu W. C. , Twu Y. C. , and Liao Y. J. , HMGCS2 Mediates Ketone Production and Regulates the Proliferation and Metastasis of Hepatocellular Carcinoma, Cancers. (2019) 11, no. 12, 10.3390/cancers11121876, 31779269. PMC6966636 31779269

[15] Tang L. , Wei R. , Chen R. , Fan G. , Zhou J. , Qi Z. , Wang K. , Wei Q. , Wei X. , and Xu X. , Establishment and Validation of a Cholesterol Metabolism-Related Prognostic Signature for Hepatocellular Carcinoma, Computational and Structural Biotechnology Journal. (2022) 20, 4402–4414, 10.1016/j.csbj.2022.07.030, 36051877. 36051877 PMC9420502

[16] Lee J. M. , Hammarén H. M. , Savitski M. M. , and Baek S. H. , Control of Protein Stability by Post-Translational Modifications, Nature Communications. (2023) 14, no. 1, 10.1038/s41467-023-35795-8, 36639369. PMC9839724 36639369

[17] Alhazzazi T. Y. , Kamarajan P. , Verdin E. , and Kapila Y. L. , Sirtuin-3 (SIRT3) and the Hallmarks of Cancer, Genes and Cancer. (2013) 4, no. 3-4, 164–171, 10.1177/1947601913486351, 2-s2.0-84887450037, 24020007. 24020007 PMC3764471

[18] Shafqat N. , Turnbull A. , Zschocke J. , Oppermann U. , and Yue W. W. , Crystal Structures of Human HMG-CoA Synthase Isoforms Provide Insights Into Inherited Ketogenesis Disorders and Inhibitor Design, Journal of Molecular Biology. (2010) 398, no. 4, 497–506, 10.1016/j.jmb.2010.03.034, 2-s2.0-77951923438, 20346956. 20346956

[19] Kim S. C. , Sprung R. , Chen Y. , Xu Y. , Ball H. , Pei J. , Cheng T. , Kho Y. , Xiao H. , Xiao L. , and Grishin N. V. , Substrate and Functional Diversity of Lysine Acetylation Revealed by a Proteomics Survey, Molecular Cell. (2006) 23, no. 4, 607–618, 10.1016/j.molcel.2006.06.026, 2-s2.0-33746992118, 16916647. 16916647

[20] Jin L. , Galonek H. , Israelian K. , Choy W. , Morrison M. , Xia Y. , Wang X. , Xu Y. , Yang Y. , Smith J. J. , Hoffmann E. , Carney D. P. , Perni R. B. , Jirousek M. R. , Bemis J. E. , Milne J. C. , Sinclair D. A. , and Westphal C. H. , Biochemical Characterization, Localization, and Tissue Distribution of the Longer Form of Mouse SIRT3, Protein Science: A Publication of the Protein Society. (2009) 18, no. 3, 514–525, 10.1002/pro.50, 2-s2.0-61449209922, 19241369. 19241369 PMC2760358

[21] Beaufrère A. , Calderaro J. , and Paradis V. , Combined Hepatocellular-Cholangiocarcinoma: An Update, Journal of Hepatology. (2021) 74, no. 5, 1212–1224, 10.1016/j.jhep.2021.01.035, 33545267. 33545267

[22] Kulthawatsiri T. , Kittirat Y. , Phetcharaburanin J. , Tomacha J. , Promraksa B. , Wangwiwatsin A. , Klanrit P. , Titapun A. , Loilome W. , and Namwat N. , Metabolomic Analyses Uncover an Inhibitory Effect of Niclosamide on Mitochondrial Membrane Potential in Cholangiocarcinoma Cells, Peer J. (2023) 11, e16512, 10.7717/peerj.16512, 38025687. 38025687 PMC10676079

[23] Lee C. L. , O′Kane G. M. , Mason W. P. , Zhang W. J. , Spiliopoulou P. , Hansen A. R. , Grant R. C. , Knox J. J. , Stockley T. L. , Zadeh G. , and Chen E. X. , Circulating Oncometabolite 2-Hydroxyglutarate as a Potential Biomarker for Isocitrate Dehydrogenase (IDH1/2) Mutant Cholangiocarcinoma, Molecular Cancer Therapeutics. (2024) 23, no. 3, 394–399, 10.1158/1535-7163.MCT-23-0460, 38015561. 38015561 PMC10911702

[24] Raggi C. , Taddei M. L. , Rae C. , Braconi C. , and Marra F. , Metabolic Reprogramming in Cholangiocarcinoma, Journal of Hepatology. (2022) 77, no. 3, 849–864, 10.1016/j.jhep.2022.04.038. 35594992

[25] Zhen Y. , Liu K. , Shi L. , Shah S. , Xu Q. , Ellis H. , Balasooriya E. R. , Kreuzer J. , Morris R. , Baldwin A. S. , Juric D. , Haas W. , and Bardeesy N. , FGFR Inhibition Blocks NF-ĸB-Dependent Glucose Metabolism and Confers Metabolic Vulnerabilities in Cholangiocarcinoma, Nature Communications. (2024) 15, no. 1, 10.1038/s41467-024-47514-y, 38714664. PMC11076599 38714664

[26] Zhang D. Y. , Zhu Y. , Wu Q. , Ma S. , Ma Y. , Shen Z. C. , Wang Z. , Sun W. , Zhou Y. C. , Wang D. , Zhou S. , Liu Z. , Kwong L. N. , and Lu Z. , USP1 Promotes Cholangiocarcinoma Progression by Deubiquitinating PARP1 to Prevent Its Proteasomal Degradation, Cell Death & Disease.(2023) 14, no. 10, 10.1038/s41419-023-06172-6, 37821462. PMC10567853 37821462

[27] Faubert B. , Solmonson A. , and DeBerardinis R. J. , Metabolic Reprogramming and Cancer progression, Science. (2020) 368, no. 6487, 10.1126/science.aaw5473, 32273439. PMC7227780 32273439

[28] Chen P. H. , Cai L. , Huffman K. , Yang C. , Kim J. , Faubert B. , Boroughs L. , Ko B. , Sudderth J. , McMillan E. A. , and Girard L. , Metabolic Diversity in Human Non-Small Cell Lung Cancer Cells, Molecular Cell. (2019) 76, no. 5, 838–851, 10.1016/j.molcel.2019.08.028, 31564558. 31564558 PMC6898782

[29] Wang Y. , Xia Y. , and Lu Z. , Metabolic Features of Cancer Cells, Cancer Communications. (2018) 38, no. 1, 10.1186/s40880-018-0335-7, 2-s2.0-85055643712, 30376896. PMC6235388 30376896

[30] Tan Y. T. , Lin J. F. , Li T. , Li J. J. , Xu R. H. , and Ju H. Q. , LncRNA‐mediated Posttranslational Modifications and Reprogramming of Energy Metabolism in Cancer, Cancer Communications. (2021) 41, no. 2, 109–120, 10.1002/cac2.12108, 33119215. 33119215 PMC7896749

[31] Grabacka M. M. , Wilk A. , Antonczyk A. , Banks P. , Walczyk-Tytko E. , Dean M. , Pierzchalska M. , and Reiss K. , Fenofibrate Induces Ketone Body Production in Melanoma and Glioblastoma Cells, Frontiers in Endocrinology. (2016) 7, 10.3389/fendo.2016.00005, 2-s2.0-84962543245, 26869992. PMC4735548 26869992

[32] Hwang C. Y. , Choe W. , Yoon K. S. , Ha J. , Kim S. S. , Yeo E. J. , and Kang I. , Molecular Mechanisms for Ketone Body Metabolism, Signaling Functions, and Therapeutic Potential in Cancer, Nutrients. (2022) 14, no. 22, 10.3390/nu14224932, 36432618. PMC9694619 36432618

[33] Geffen Y. , Anand S. , Akiyama Y. , Yaron T. M. , Song Y. , Johnson J. L. , Govindan A. , Babur Ö. , Li Y. , Huntsman E. , Wang L. B. , Birger C. , David H. , Zhang Q. , Miller M. , Maruvka Y. E. , Haradhvala N. J. , Calinawan A. , Belkin S. , Kerelsky A. , Clauser K. R. , Krug K. , Satpathy S. , Payne S. H. , Mani D. R. , Gillette M. A. , Dhanasekaran S. M. , Thiagarajan M. , Mesri M. , Rodriguez H. , Ana R. , Carr S. A. , Lazar A. J. , Aguet F. , Cantley L. C. , Ding L. , Getz G. , and Clinical Proteomic Tumor Analysis Consortium , Pan-Cancer Analysis of Post-Translational Modifications Reveals Shared Patterns of Protein Regulation, Cell. (2023) 186, no. 18, 3945–3967, 10.1016/j.cell.2023.07.013, 37582358. 37582358 PMC10680287

[34] Shimazu T. , Hirschey M. D. , Hua L. , Dittenhafer-Reed K. E. , Schwer B. , Lombard D. B. , Li Y. , Bunkenborg J. , Alt F. W. , Denu J. M. , Jacobson M. P. , and Verdin E. , SIRT3 Deacetylates Mitochondrial 3-Hydroxy-3-Methylglutaryl CoA Synthase 2 and Regulates Ketone Body Production, Cell Metabolism. (2010) 12, no. 6, 654–661, 10.1016/j.cmet.2010.11.003, 2-s2.0-78649509214, 21109197. 21109197 PMC3310379

[35] Hwang S. , Park S. , Kim J. H. , Bang S. B. , Kim H. J. , Ka N. L. , Ko Y. , Kim S. S. , Lim G. Y. , Lee S. , Shin Y. K. , Park S. Y. , Kim S. , and Lee M. O. , Targeting HMG-CoA Synthase 2 Suppresses Tamoxifen-Resistant Breast Cancer Growth by Augmenting Mitochondrial Oxidative Stress-Mediated Cell Death, Life Sciences. (2023) 328, 121827, 10.1016/j.lfs.2023.121827, 37276910. 37276910

[36] Neuwirt H. , Bouchal J. , Kharaishvili G. , Ploner C. , Jöhrer K. , Pitterl F. , Weber A. , Klocker H. , and Eder I. E. , Cancer-Associated Fibroblasts Promote Prostate Tumor Growth and Progression Through Upregulation of Cholesterol and Steroid Biosynthesis, Cell Communication and Signaling. (2020) 18, no. 1, 10.1186/s12964-019-0505-5, 31980029. PMC6979368 31980029

[37] Tang H. , Wu Y. , Qin Y. , Wang H. , Jia Y. , Yang S. , Luo S. , and Wang Q. , Predictive Significance of HMGCS2 for Prognosis in Resected Chinese Esophageal Squamous Cell Carcinoma Patients, Onco Targets and therapy. (2017) 10, 2553–2560, 10.2147/OTT.S132543, 2-s2.0-85019556991, 28546759. PMC5438074 28546759

